# Association between stress hyperglycemia ratio and diabetes mellitus mortality in American adults: a retrospective cohort study and predictive model establishment based on machine learning algorithms (NHANES 2009–2018)

**DOI:** 10.1186/s13098-024-01324-w

**Published:** 2024-04-02

**Authors:** Fengjuan Yan, Limin Zhao, Xiaoqing Quan, Jialiang Zhu

**Affiliations:** 1 Department of Geriatrics, Shenzhen Longhua District Central Hospital, Shenzhen, Guangdong China; 2 The First Affiliated Hospital of Jinan University, Guangzhou, Guangdong China

**Keywords:** Stress hyperglycemia ratio, NHANES, Machine learning algorithm, Retrospective cohort, Mediating effect

## Abstract

**Background:**

Stress hyperglycemia is a physiological response of the body under stress to make adaptive adjustments in response to changes in the internal environment. The stress hyperglycemia ratio (SHR) is a new indicator after adjusting the basal blood glucose level of the population. Previous studies have shown that SHR is associated with poor prognosis in many diseases, such as cardiovascular and cerebrovascular diseases and delirium in elderly patients. However, there are currently no studies on the correlation between SHR and the general U.S. population. The purpose of this study was to examine the association between SHR and adverse outcomes among adults in the United States in general.

**Methods:**

Data on 13,315 follow-up cohorts were extracted from NHANES. The study population was divided into four groups according to quartiles of SHR. The primary outcomes were all-cause mortality and diabetes mellitus mortality. The relationship between SHR and outcomes was explored using restricted cubic splines, COX proportional hazards regression, Kaplan-Meier curves, and mediation effects. SHR is incorporated into eight machine learning algorithms to establish a prediction model and verify the prediction performance.

**Results:**

A total of 13,315 individual data were included in this study. Restricted cubic splines demonstrated a “U-shaped” association between SHR and all-cause mortality and diabetes mellitus mortality, indicating that increasing SHR is associated with an increased risk of adverse events. Compared with lower SHR, higher SHR was significantly associated with an increased risk of all cause mortality and diabetes mellitus mortality (HR > 1, *P* < 0.05). The mediating effect results showed that the positively mediated variables were segmented neutrophils and aspartate aminotransferase, and the negatively mediated variables were hemoglobin, red blood cell count, albumin, and alanine aminotransferase. The ROC of the eight machine learning algorithm models are XGBoost (0.8688), DT (0.8512), KNN (0.7966), RF (0.8417), Logistic regression (0.8633), ENET (0.8626), SVM (0.8327) and MLP (0.8662).

**Conclusion:**

SHR can be used as a predictor of all cause mortality and diabetes mellitus mortality in the general adult population in the United States. Higher SHR is significantly associated with an increased risk of poor prognosis, especially in those aged < 65 years and in women.

**Supplementary Information:**

The online version contains supplementary material available at 10.1186/s13098-024-01324-w.

## Introduction

Stress hyperglycemia occurs when the body responds physiologically to stress or severe illness, mediated by the interplay or coordination of catecholamines, growth hormone, cortisol, and cytokines [1–4]. Newly developed hyperglycemia patients exhibit a higher mortality rate compared to those with previously diagnosed hyperglycemia or known diabetes [5–6], indicating differential effects of chronic glucose levels on the relationship between admission glucose and mortality. Possible explanations for this phenomenon include, Firstly, patients with new-onset hyperglycemia may be in the early stages of the disease, and the disease has not yet been effectively controlled, leading to worsening of the condition and increased risk of death. Secondly, patients with new-onset hyperglycemia may not have received the same level of treatment as patients with known hyperglycemia or diabetes, which may lead to increased mortality due to delays in seeking medical treatment or failure to receive appropriate treatment in a timely manner. Thirdly, patients with new-onset hyperglycem i.a. may have other underlying health problems before the disease is diagnosed, and these problems may increase the risk of death. Glycated hemoglobin (HbA1c) is a classic marker used to assess the average blood glucose concentration over the past 8–12 weeks, accurately reflecting the level of chronic glucose over time. To mitigate the influence of background glucose, researchers have introduced HbA1c as a baseline glucose level when assessing stress hyperglycemia, proposing the stress hyperglycemia ratio (SHR) as a new indicator to evaluate acute hyperglycemia more accurately [7].

In terms of the correlation between SHR and cardiovascular diseases, a cohort analysis involving 2290 emergency patients undergoing treatment showed that in univariate analysis, for every 0.1 increase in SHR, the risk increased by 23%; after adjusting for demographic variables, the risk increased by 20%, both differences being significant [7]. Further studies indicate that SHR is independently associated with short-term and long-term adverse outcomes in acute coronary syndrome (ACS) and short-term adverse outcomes in acute myocardial infarction (AMI) [8–13]. A study involving 2875 Chinese adults with type 2 diabetes and heart failure found that both higher and lower SHR patients had poor prognoses [14]. Regarding the correlation between SHR and cerebrovascular diseases, a two-center prospective study showed that after adjusting for covariates, for each 1-unit increase in SHR, the risk of early hematoma expansion in spontaneous intracerebral hemorrhage (ICH) patients increased by 16.535 times (95% CI: 3.572–76.543, *p* < 0.001), indicating an independent correlation between SHR and early hematoma expansion in ICH patients. The predictive model incorporating SHR had an Area Under Curve (AUC) of 0.759 (0.694–0.825), suggesting that SHR is a good predictor of early hematoma expansion in ICH patients [15]. Additionally, SHR is independently associated with increased risk of delirium and short-term mortality in critically ill patients after esophagectomy [16–17].

However, there is currently no relevant research on the association between SHR and all cause mortality risk or diabetes mellitus mortality risk in the general population. Therefore, we aim to investigate the relationship between SHR and the risk of all cause mortality and diabetes mellitus mortality in the general population, as well as its predictive value, using data from the National Health and Nutrition Examination Survey (NHANES) from 2009 to 2018.

## Methods

### Data source

The data for this cohort study are sourced from the NHANES 2009–2018, comprising 49,693 American participants aged 18 to 100 years old. NHANES conducts surveys on nationally representative samples, collecting extensive data on individual health, nutrition intake, lifestyle, and environmental factors (https://wwwn.cdc.gov/nchs/nhanes/Default.aspx), primarily aimed at assessing the health and nutritional status of American adults and children. These data are used for studying the epidemiological characteristics of chronic diseases, nutritional deficiencies, and the effectiveness of health policy formulation and implementation, among other purposes. The National Center for Health Statistics (NCHS) Ethics Review Board approved the NHANES research program. All study participants provided written informed consent. As of December 31, 2019, overall mortality and diabetes mellitus mortality were determined through linkage with the National Death Index. The stress hyperglycemia ratio (SHR) is defined as an index calculated using the following formula: SHR = (admission blood glucose) (mmol/L) / (1.59 * HbA1c [%] − 2.59).

### Exclusion criteria

1. Individuals younger than 18 years old or older than 100 years old.
2. Individuals with missing values for HbA1c and fasting blood glucose.

### Research variable

The study variables include: age, gender, body mass index (BMI), race, smoking status, coronary heart disease, congestive heart failure, cancer or malignancy, emphysema, stroke, SHR, white blood cell count (WBC), red blood cell count (RBC), hemoglobin, platelet count (PLT), segmented neutrophils number (NEU), monocyte number, albumin, sodium, creatinine, potassium, blood urea nitrogen (BUN), alanine aminotransferase (ALT), and aspartate aminotransferase (AST). Race/ethnicity categories include Mexican American, Non-Hispanic Black, Non-Hispanic White, Other Hispanic, and Other Race - Including Multi-Racial. Smoking is categorized into four groups: Every day, Some days, Not at all, and Other (Refused, Don’t know, Missing).

### Outcome

1. All-cause mortality.
2. Diabetes mellitus mortality.

### Statistical analysis

Variables with missing values exceeding 20% will be excluded, while variables with missing values below 20% will be imputed using multiple imputation methods. Variance inflation factor (VIF) will be used to assess multicollinearity among variables. Variables with VIF exceeding 5 will be removed from the study to address multicollinearity issues. Patients will be divided into 4 groups based on the quartiles of SHR. Continuous variables following a normal distribution will be presented as mean (standard deviation [sd]) and analyzed using analysis of variance (ANOVA). Categorical variables will be presented as numbers and percentages, and analyzed using either the χ^2 test or Fisher’s exact test.

Kaplan-Meier (K-M) curves were utilized to assess the survival probabilities of four groups of patients and inter-group differences were evaluated through log-rank tests. Proportional hazards regression models (Cox regression models) were employed to assess the risk ratio of event occurrence, expressed as hazard ratios (HR) and 95% confidence intervals (95% CI). Model I did not adjust covariates, while Model II included all covariates for adjustment. Cox regression models with restricted cubic splines (RCS) were utilized to examine potential non-linear relationships between SHR changes and outcome events.

### Subgroup Analysis

We also conducted a subgroup analysis based on pre-specified age and gender. Patients were stratified into two groups based on age (< 65 years and ≥ 65 years), and baseline characteristics of comorbidities were presented. Cox proportional hazards regression analysis was performed for each subgroup.

### Establishment and validation of Prediction models

The dataset was randomly divided into training and validation sets in a 7:3 ratio. To ensure the robustness of the model, five-fold cross-validation was conducted on the training set for iterative testing and tuning to determine hyperparameters and generate the optimal model. We performed multivariable Cox regression analysis on variables. For rapid prediction, only demographic characteristics, comorbidities, and SHR variables were included with a significance level of *P* < 0.1, while other invasively obtained blood indicators were excluded. Selected variables were analyzed using Logistic Regression (LR), Decision Tree (DT), K-nearest Neighbors (KNN), Random Forest (RF), Extreme Gradient Boosting (XGBoost), Elastic Net (ENET), Support Vector Machine (SVM), and Multilayer Perceptron (MLP) algorithms.

The training set was used to establish models to predict all-cause mortality risk, while the testing set was utilized to evaluate the effectiveness of the models. The area under the receiver operating characteristic curve (AUC) of the receiver operating characteristic curve (ROC) was used to determine the model’s performance. Decision Curve Analysis (DCA) was employed to assess clinical effectiveness, and calibration curves were used to judge the accuracy of absolute risk prediction. To enhance predictive efficiency and clinical utility, the model with the best performance was used to develop an online risk calculator.

### Mediation analysis

Investigate the potential mediating effect of all covariates, except for gender and ethnicity, on the relationship between SHR and all-cause mortality. This study aims to assess the direct and indirect effects of each mediator and determine the proportion of the total effect mediated by each covariate. Survival analysis was conducted using the survreg method with Bootstrap modeling and 500 simulations. The proportion value of SHR on all-cause mortality is used to determine how much of the effect of SHR is mediated by covariates.

A two-tailed *P* < 0.05 was considered statistically significant. Statistical analysis was performed using R software (version 4.3.1).

## Results

### Baseline characteristics

**Fig. 1:**
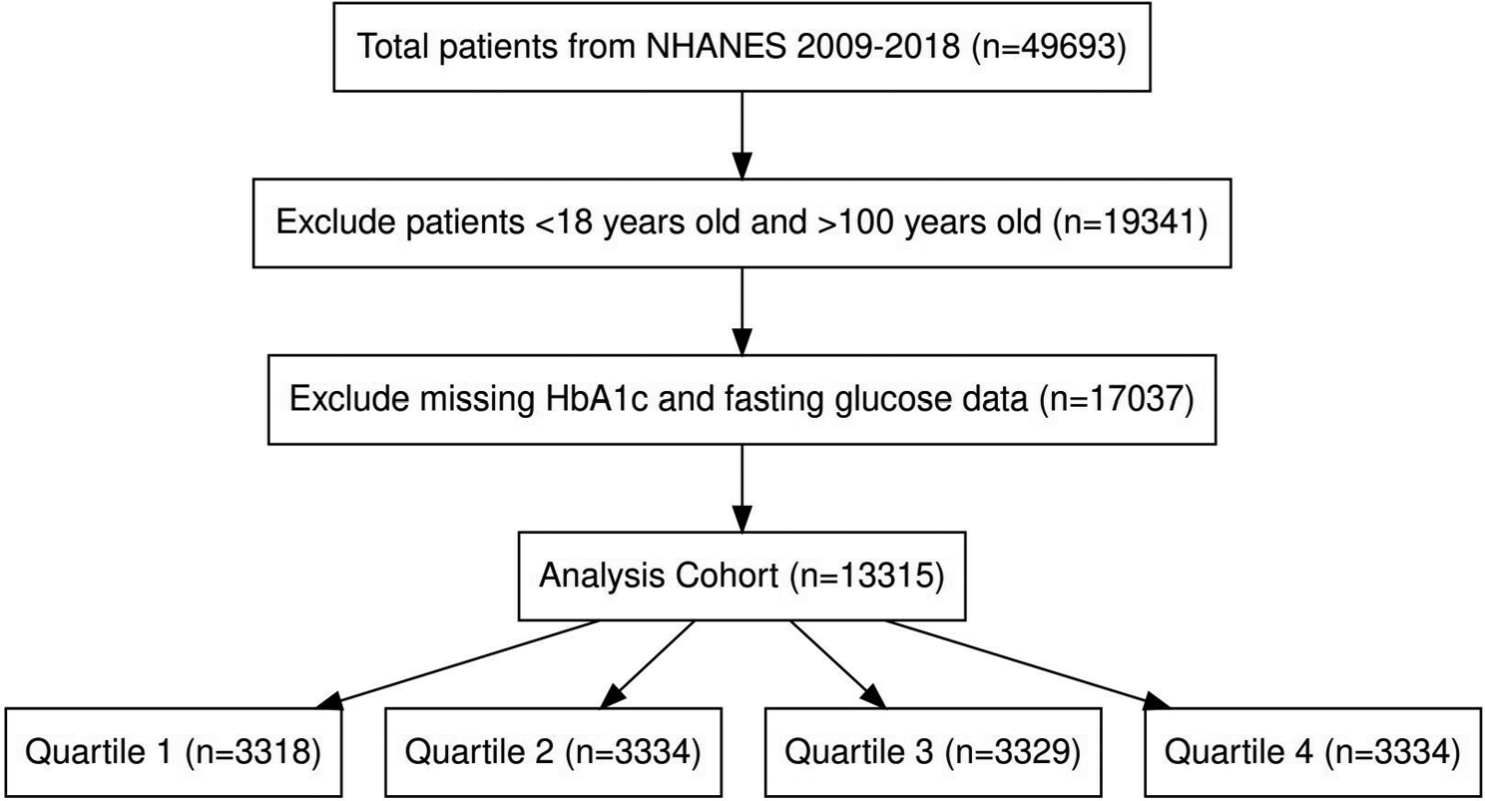
Selection of study population from NHANES (2009–2018)

As shown in Fig. 1, a total of 13,315 people met the criteria. The maximum follow-up period was 132 months. Figure S1 shows the missing proportion of each variable, and Table S1 shows the variance inflation factor of each variable, indicating that there is no multicollinearity among the variables. Of the total population, 6,433 (48%) were male, with the largest proportion of non-Hispanic whites (5,251 (39%)), 438 (3%) had congestive heart failure, and 533 (4%) had coronary heart disease, 497 (4%) had stroke, 254 (2%) had Emphysema, and 1195 (9%) had Cancer or malignancy. According to the SHR quartiles, the number of patients was divided into four equal parts, namely Quartile 1 (0.109 ≤ SHR < 0.839), Quartile 2 (0.839 ≤ SHR < 0.906), Quartile 3 (0.906 ≤ SHR < 0.986), and Quartile 4 (0.986). ≤ SHR ≤ 2.79). Table 1 shows the baseline characteristics of the study subjects. Patients in Quartile 4 had higher BMI, NEU, Hemoglobin, RBC, WBC, BUN, Creatinine, Albumin, AST, and ALT, and had lower PLT.

**Table 1:** Patient demographics and baseline characteristics

Characteristic (Mean ± SD)	SHR					p-value
	Overall, *N* = 13,315	[0.109,0.839), *N* = 3,318	[0.839,0.906), *N* = 3,334	[0.906,0.986), *N* = 3,329	[0.986,2.79], *N* = 3,334	
**Age (years)**	48 ± 18	51 ± 18	48 ± 18	47 ± 18	49 ± 19	< 0.001
BMI	29 ± 7	29 ± 7	29 ± 7	29 ± 7	30 ± 7	< 0.001
Gender						< 0.001
Male	6,433 (48%)	1,340 (40%)	1,444 (43%)	1,688 (51%)	1,961 (59%)	
Female	6,882 (52%)	1,978 (60%)	1,890 (57%)	1,641 (49%)	1,373 (41%)	
Race						< 0.001
Mexican American	2,013 (15%)	409 (12%)	476 (14%)	589 (18%)	539 (16%)	
Non-Hispanic Black	1,434 (11%)	339 (10%)	384 (12%)	352 (11%)	359 (11%)	
Non-Hispanic White	5,251 (39%)	1,035 (31%)	1,320 (40%)	1,393 (42%)	1,503 (45%)	
Other Hispanic	2,753 (21%)	1,087 (33%)	655 (20%)	490 (15%)	521 (16%)	
Other Race - Including Multi-Racial	1,864 (14%)	448 (14%)	499 (15%)	505 (15%)	412 (12%)	
Smoke						< 0.001
Every day	2,034 (15%)	576 (17%)	518 (16%)	482 (14%)	458 (14%)	
Some days	502 (4%)	114 (3%)	122 (4%)	123 (4%)	143 (4%)	
Not at all	3,050 (23%)	696 (21%)	697 (21%)	743 (22%)	914 (27%)	
Other	7,729 (58%)	1,932 (58%)	1,997 (60%)	1,981 (60%)	1,819 (55%)	
Monocyte (1000 cells/uL)	0.53 ± 0.21	0.54 ± 0.19	0.53 ± 0.18	0.53 ± 0.25	0.54 ± 0.19	0.138
NEU (1000 cell/uL)	3.98 ± 1.77	3.90 ± 2.16	3.91 ± 1.64	3.97 ± 1.56	4.14 ± 1.66	< 0.001
Hemoglobin (g/dL)	14.06 ± 1.54	13.58 ± 1.52	13.97 ± 1.45	14.26 ± 1.44	14.42 ± 1.59	< 0.001
PLT (1000 cells/uL)	236 ± 62	240 ± 66	239 ± 62	235 ± 60	229 ± 62	< 0.001
RBC (million cells/uL)	4.69 ± 0.51	4.62 ± 0.52	4.68 ± 0.50	4.73 ± 0.49	4.74 ± 0.53	< 0.001
WBC (1000 cells/uL)	6.82 ± 2.48	6.77 ± 2.65	6.80 ± 2.89	6.81 ± 2.19	6.92 ± 2.09	0.064
BUN (mmol/L)	4.89 ± 2.13	4.96 ± 2.33	4.75 ± 1.97	4.80 ± 1.87	5.04 ± 2.29	< 0.001
Creatinine (umol/L)	79 ± 42	81 ± 50	76 ± 31	77 ± 39	81 ± 46	< 0.001
Albumin (g/dL)	4.21 ± 0.35	4.14 ± 0.36	4.20 ± 0.33	4.24 ± 0.33	4.24 ± 0.37	< 0.001
AST (U/L)	25 ± 19	25 ± 20	25 ± 24	25 ± 14	26 ± 17	0.019
ALT (U/L)	25 ± 18	23 ± 16	24 ± 17	25 ± 19	27 ± 20	< 0.001
Potassium (mmol/L)	4.03 ± 0.35	4.03 ± 0.36	4.03 ± 0.34	4.02 ± 0.34	4.02 ± 0.36	0.779
Sodium (mmol/L)	139.45 ± 2.32	139.44 ± 2.32	139.52 ± 2.25	139.49 ± 2.24	139.34 ± 2.46	0.007
Congestive heart failure						< 0.001
Yes	438 (3%)	130 (4%)	94 (3%)	84 (3%)	130 (4%)	
No	12,877 (97%)	3,188 (96%)	3,240 (97%)	3,245 (97%)	3,204 (96%)	
Coronary heart disease						0.001
Yes	533 (4%)	153 (5%)	103 (3%)	120 (4%)	157 (5%)	
No	12,782 (96%)	3,165 (95%)	3,231 (97%)	3,209 (96%)	3,177 (95%)	
Stroke						0.005
Yes	497 (4%)	140 (4%)	118 (4%)	95 (3%)	144 (4%)	
No	12,818 (96%)	3,178 (96%)	3,216 (96%)	3,234 (97%)	3,190 (96%)	
Emphysema						0.296
Yes	254 (2%)	74 (2%)	58 (2%)	55 (2%)	67 (2%)	
No	13,061 (98%)	3,244 (98%)	3,276 (98%)	3,274 (98%)	3,267 (98%)	
Cancer or malignancy						0.149
Yes	1,195 (9%)	315 (9%)	290 (9%)	272 (8%)	318 (10%)	
No	12,120 (91%)	3,003 (91%)	3,044 (91%)	3,057 (92%)	3,016 (90%)	

### Restricted cubic splines

**Fig. 2:**
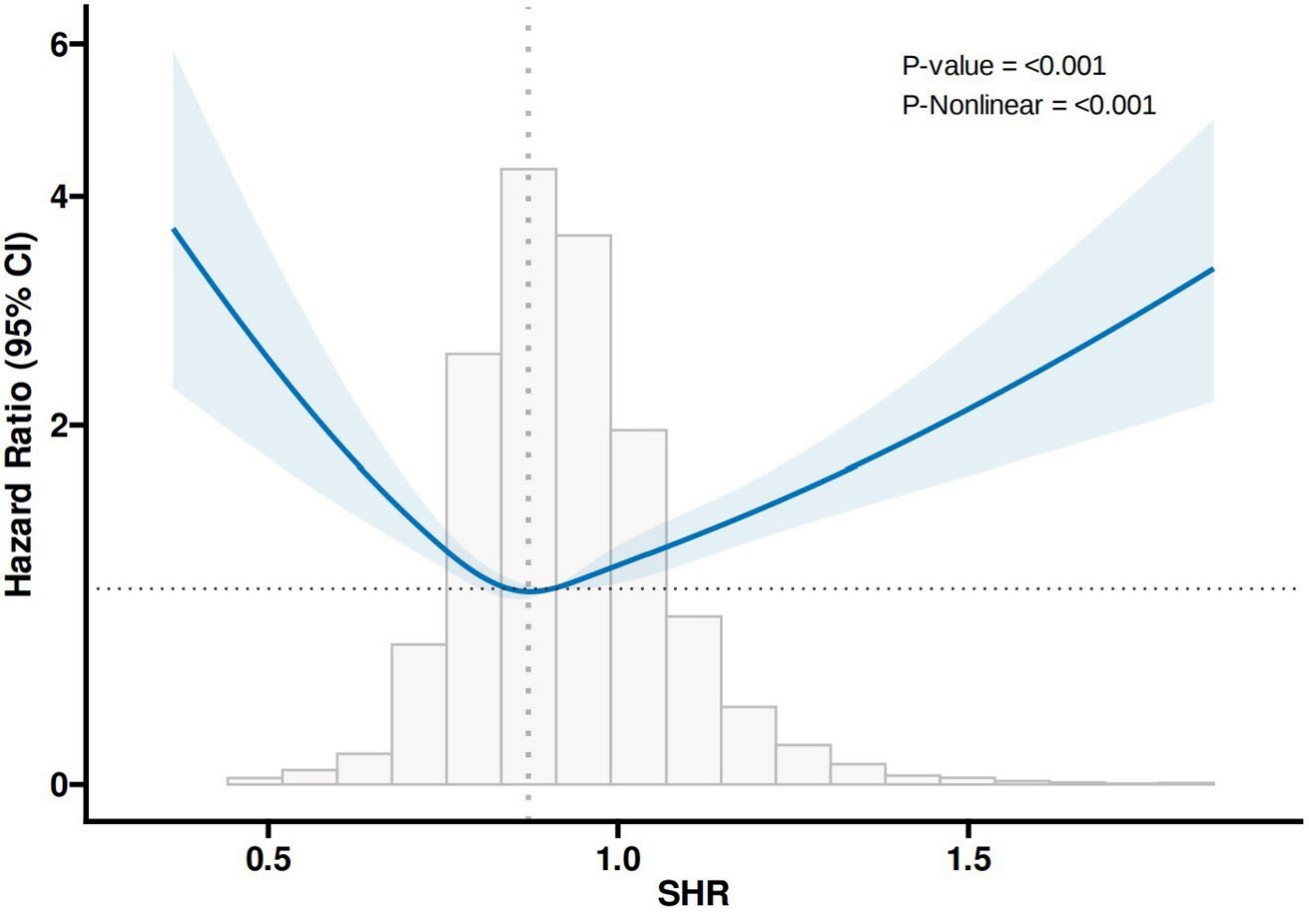
RCS results for all-cause mortality

**Fig. 3:**
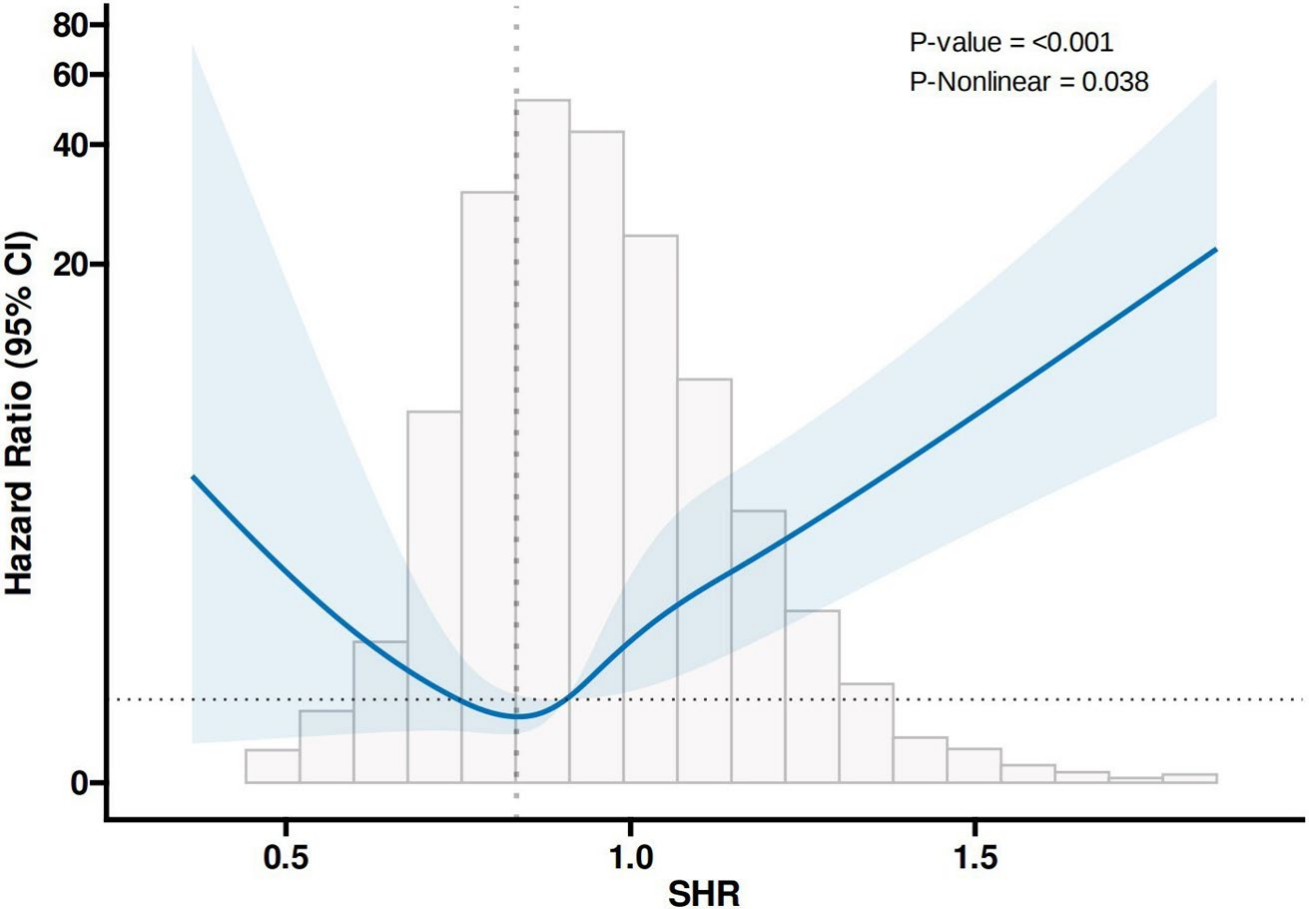
RCS results for diabetes mellitus mortality

In the analyses of all-cause mortality (Fig. 2) and diabetes mellitus mortality (Fig. 3) events, RCS analysis adjusted for the effects of gender, age, ethnicity, and BMI revealed a “U-shaped” association between SHR and the risk of outcome events. The inflection points of the RCS curves were at SHR = 0.87 and SHR = 0.83, both in Quartile 2, representing the turning points in the relationship between SHR and the occurrence of outcome events. Therefore, Quartile 2 was defined as the reference category.

### Clinical outcomes

**Fig. 4:**
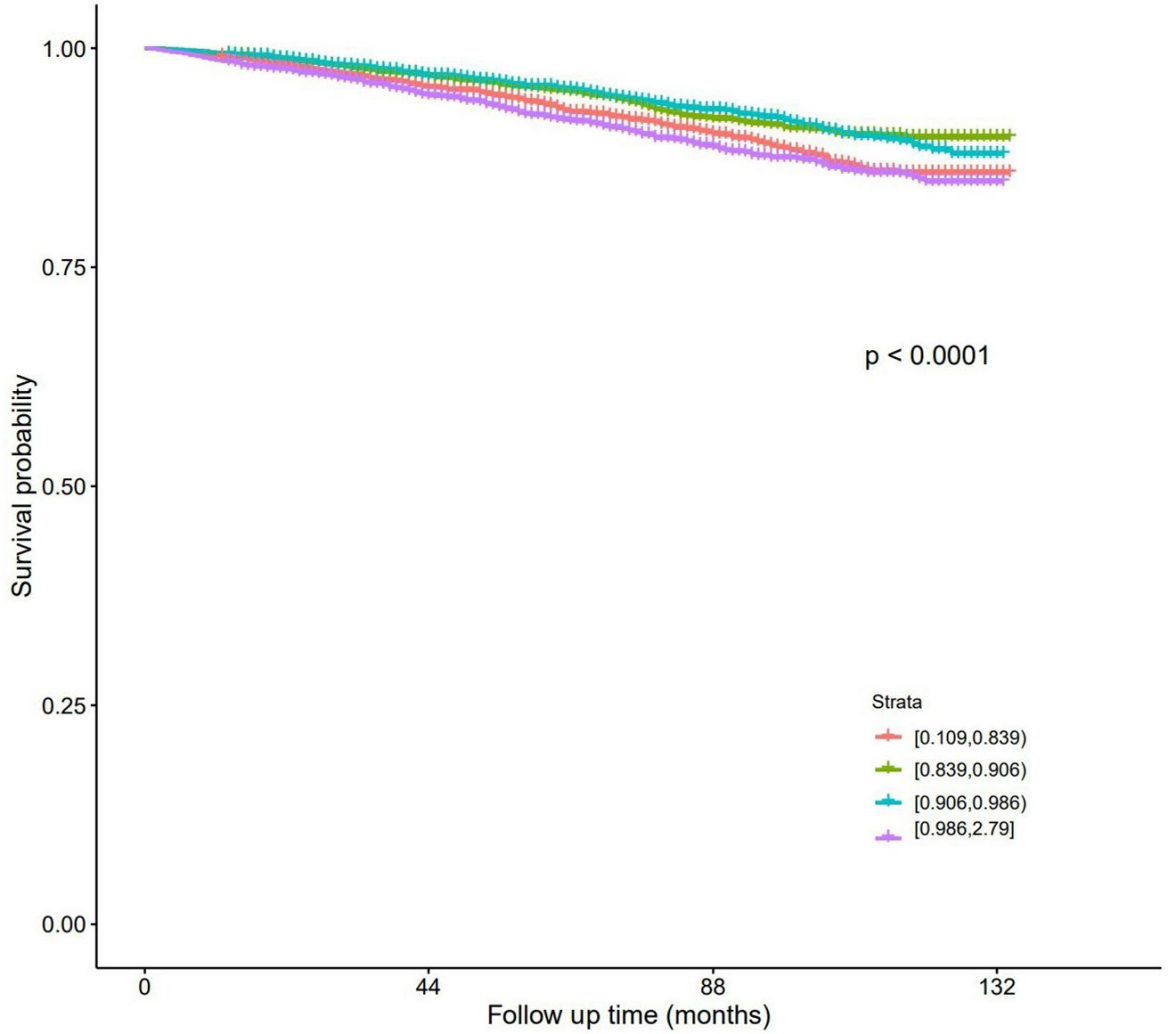
K-M survival curve (All-cause mortality)

**Fig. 5:**
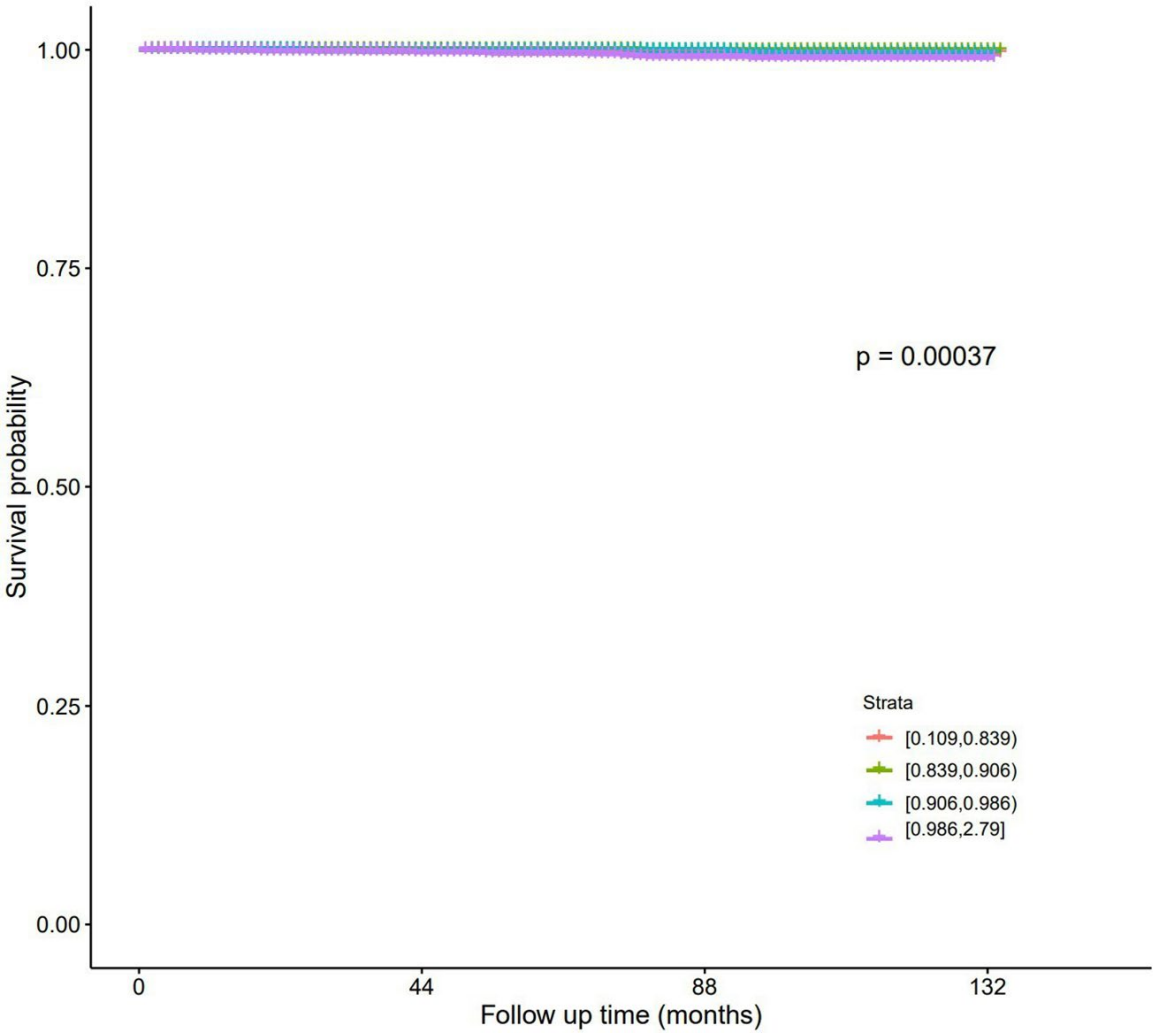
K-M survival curve (Diabetes mellitus mortality)

The number of all-cause mortality for Quartiles 1–4 were 293, 208, 198, and 288 respectively, while the number of diabetes mellitus mortality were 8, 2, 7, and 18 (Table 2). For all-cause mortality (Table 3), with Quartile 2 as the reference, in Model I, the HR (95% CI) for Quartiles 1, 3, and 4 were 1.36 (1.14–1.63), 0.99 (0.82–1.20), and 1.54 (1.29–1.84) respectively. In Model II, the corresponding values were 0.98 (0.82–1.17), 0.95 (0.78–1.15), and 1.24 (1.03–1.49), with Quartile 4 showing significant differences. For diabetes mellitus mortality (Table 4), with Quartile 2 as the reference, in Model I, the HR (95% CI) for Quartiles 1, 3, and 4 were 3.84 (0.82–18.1), 3.65 (0.76–17.6), and 10.1 (2.34–43.6) respectively. In Model II, the corresponding values were 2.42 (0.50–11.7), 3.10 (0.64–15.1), and 5.66 (1.28–25.0), with Quartile 4 showing significant differences. K-M curves show that in all-cause mortality (Fig. 4) and diabetes mellitus mortality (Fig. 5), patients in Quartile 4 had the lowest survival probabilities, and the differences were significant.

**Table 2:** All-cause mortality and diabetes mellitus mortality

SHR		[0.109,0.839)	[0.839,0.906)	[0.906,0.986)	[0.986,2.79]	P Value
Mortality, n (%)						
All-cause mortality	293 (8.8%)		208 (6.2%)	198 (5.9%)	288 (8.6%)	< 0.001
Diabetes mellitus mortality	8 (0.24%)		2 (0.06%)	7 (0.21%)	18 (0.54%)	0.001

**Table 3:** COX regression model (All-cause mortality)

SHR	Unadjusted HR (95%CI)	P Value	Adjusted HR (95%CI)	P Value
[0.839,0.906)	Reference		Reference	
[0.109,0.839)	1.36 (1.14–1.63)	<0.001	0.98 (0.82–1.17)	0.83
[0.906,0.986)	0.99 (0.82–1.20)	0.93	0.95 (0.78–1.15)	0.58
[0.986,2.79]	1.54 (1.29–1.84)	<0.001	1.24 (1.03–1.49)	0.023

**Table 4:** COX regression model (Diabetes mellitus mortality)

SHR	Unadjusted HR (95%CI)	P Value	Adjusted HR (95%CI)	P Value
[0.839,0.906)	Reference		Reference	
[0.109,0.839)	3.84 (0.82–18.1)	0.089	2.42 (0.50–11.7)	0.27
[0.906,0.986)	3.65 (0.76–17.6)	0.11	3.10 (0.64–15.1)	0.16
[0.986,2.79]	10.1(2.34–43.6)	0.002	5.66 (1.28–25.0)	0.022

### Subgroup Analysis

Table 5 presents the results of subgroup analysis for all-cause mortality. In the Age < 65 and female group, Quartile 4 showed a higher risk of death regardless of covariate adjustment, while no differences were observed in the Age ≥ 65 and male groups. Table 6 presents the results of subgroup analysis for diabetes mellitus mortality. In the Age < 65, male, and female groups, Quartile 4 exhibited a higher risk of death regardless of covariate adjustment. Table S2 shows the incidence rates of comorbidities in the population grouped by age. Individuals aged ≥ 65 years had higher rates of congestive heart failure, coronary heart disease, stroke, emphysema, and cancer or malignancy.

**Table 5:** Subgroup analysis for all-cause mortality

SHR	Number	Unadj-HR (95%CI)	Unadj-P value	Adj-HR (95%CI)	Adj-P value
Age<65	10,233				
[0.839,0.906)		Reference		Reference	
[0.109,0.839)		1.34 (0.96–1.87)	0.09	1.16 (0.82–1.64)	0.4
[0.906,0.986)		0.91 (0.63–1.32)	0.63	0.98 (0.67–1.42)	0.9
[0.986,2.79]		1.74 (1.26–2.41)	<0.001	1.52 (1.08–2.14)	0.015
Age ≥ 65	3082				
[0.839,0.906)		Reference		Reference	
[0.109,0.839)		1.16 (0.94–1.43)	0.17	0.94 (0.76–1.16)	0.57
[0.906,0.986)		1.05 (0.83–1.32)	0.7	0.90 (0.71–1.14)	0.38
[0.986,2.79]		1.34 (1.08–1.66)	0.007	1.14 (0.92–1.43)	0.24
Female	6882				
[0.839,0.906)		Reference		Reference	
[0.109,0.839)		1.29 (0.99–1.68)	0.059	0.96 (0.74–1.26)	0.78
[0.906,0.986)		0.86 (0.63–1.18)	0.36	0.93 (0.68–1.28)	0.67
[0.986,2.79]		2.01 (1.53–2.65)	<0.001	1.46 (1.10–1.95)	0.01
Male	6433				
[0.839,0.906)		Reference		Reference	
[0.109,0.839)		1.49 (1.17–1.89)	0.001	1.03 (0.81–1.31)	0.81
[0.906,0.986)		1.01 (0.79–1.29)	0.95	0.94 (0.73–1.21)	0.62
[0.986,2.79]		1.18 (0.93–1.49)	0.17	1.10 (0.86–1.40)	0.45

**Table 6:** Subgroup analysis for diabetes mellitus mortality

SHR	Number	Unadj-HR (95%CI)	Unadj-P value	Adj-HR (95%CI)	Adj-P value
Age<65	10,233				
[0.839,0.906)		Reference		Reference	
[0.109,0.839)		4.07 (0.45–36.4)	0.21	2.34 (0.24–22.6)	0.46
[0.906,0.986)		4.13 (0.46–36.9)	0.2	3.45 (0.38–31.7)	0.27
[0.986,2.79]		12.5 (1.61–96.5)	0.016	8.2 (1.02–66.0)	0.048
Age ≥ 65	3082				
[0.839,0.906)		Reference		Reference	
[0.109,0.839)		3.07 (0.34–27.5)	0.32	3.15 (0.28–35.5)	0.35
[0.906,0.986)		3.14 (0.33–30.1)	0.32	3.19 (0.27–38.3)	0.36
[0.986,2.79]		7.09 (0.87–57.7)	0.067	3.78 (0.41–35.1)	0.24
Female	6882				
[0.839,0.906)		Reference		Reference	
[0.109,0.839)		2.65 (0.28–25.5)	0.4	1.45 (0.40–5.27)	0.58
[0.906,0.986)		1.2 (0.07–19.1)	0.9	0.86 (0.11–6.67)	0.89
[0.986,2.79]		12.4 (1.56–99.5)	0.017	5.51 (1.79–16.9)	0.003
Male	6433				
[0.839,0.906)		Reference		Reference	
[0.109,0.839)		5.38 (0.63–46)	0.12	3.94 (1.45–10.8)	0.007
[0.906,0.986)		5.36 (0.65–44.6)	0.12	4.9 (1.91–12.6)	<0.001
[0.986,2.79]		8.19 (1.05–64)	0.045	6.64 (2.86–15.4)	<0.001

### Establishment and validation of the Prediction Mode

**Fig. 6:**
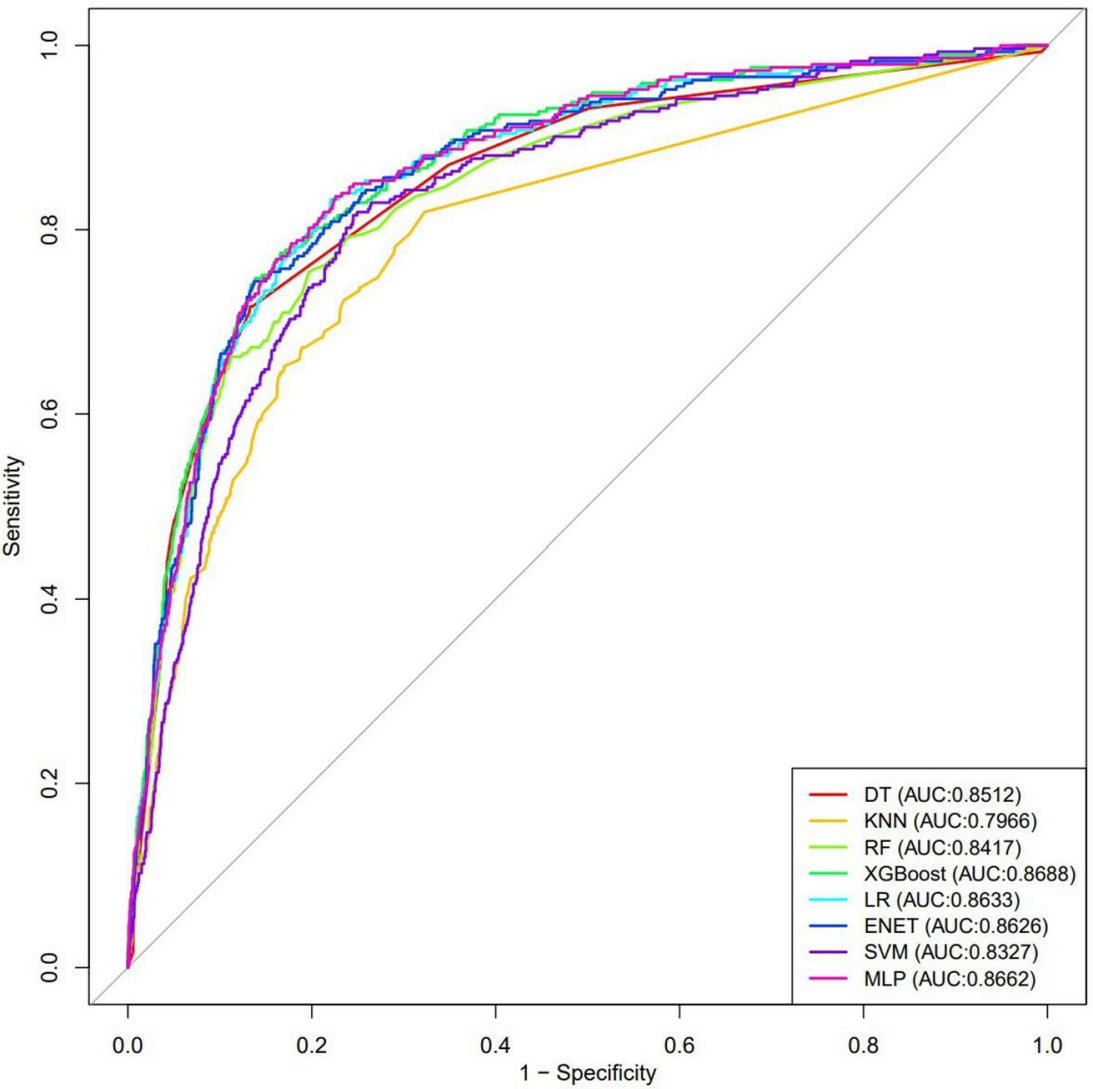
ROC curves of eight machine learning algorithm models

Variables with a P-value < 0.05 in the univariate analysis were included in the multivariable analysis. The results of the multivariable analysis can be found in Table S3. Based on the principle of simplicity in inquiry, the variables SHR, Age, Smoking status, Gender, Race, Congestive heart failure, Coronary heart disease, Stroke, and Cancer or malignancy were included in the eight machine learning algorithms models. Figure 6 shows the ROC curves of each model. XGBoost has the largest AUC value (0.8688). The AUC values of other models are DT (0.8512), KNN (0.7966), RF (0.8417), Logistic regression (0.8633), and ENET (0.8626). ), SVM (0.8327), MLP (0.8662). Figure S2 shows the calibration curve of each model, and the calibration curve of the XGBoost model does not deviate significantly from the reference line, indicating that it has good predictive performance. According to the DCA curve (Figure S3), the XGBoost model showed greater net benefit, indicating that XGBoost has good clinical effectiveness.

In order to facilitate the use of clinicians and researchers, we used the Shiny platform to develop a web application based on the XGBoost model (https://shrpmci.shinyapps.io/xgboost/). The clinical characteristics of the new sample can be entered in the corresponding location of the web interface. The web application can then help predict the 132-month risk of all-cause mortality based on the individual’s information.

### Mediation analysis

The mediation analysis results show that SHR has a significant overall effect on survival, with coefficients and p-values as shown in Table S4. The range of mediation proportions for each covariate ranges from negative values to 23.1%. Covariates exhibiting positive indirect mediation include segmented neutrophils and AST, with mediation effects of 6.3 (3.0, 46.7) and 1.5 (0.1, 7.9) respectively. Covariates exhibiting negative mediation include hemoglobin, RBC, albumin, and ALT, with mediation effects of -69.3 (-248.7, -34.1), -36.1 (-171.6, -15.3), -44.4 (-181.8, -20.4), and − 19.4 (-74.9, -6.9) respectively.

## Discussion

In our study, we conducted a retrospective analysis of tracking data from 13,315 American adults over five survey cycles, with a median follow-up period of 71 months. The results indicate that regardless of covariate adjustment, the rates of all-cause mortality and diabetes-related mortality were significantly higher in the highest quartile of SHR (Quartile 4) compared to Quartile 2 (the interval where the lowest point of RCS lies). The risk of both all-cause mortality and diabetes-related mortality increased when SHR exceeded 0.87, particularly in the population aged < 65 years and among females. In addition, eight machine learning algorithms were used to establish models for variables including SHR, and the AUC of seven of the models exceeded 0.83, indicating good predictive performance. To our knowledge, this is the first study to explore the relationship between SHR and all-cause mortality as well as diabetes mellitus mortality in the general adult population of the United States.

Previous retrospective studies and cross-sectional studies have investigated the independent correlation between SHR and adverse outcomes in various diseases such as ACS, AMI, delirium in the elderly, and critically ill patients, among others [13, 16, 18]. Higher SHR has been associated with an increased risk of adverse outcomes. A retrospective study involving 4,362 subjects who underwent PCI with a median follow-up of 2.5 years found that compared to the lowest quartile of SHR, the highest quartile had a risk ratio of 1.31 (95% CI 1.05–1.64) for experiencing major adverse cardiovascular and cerebrovascular events (MACCE) [11]. In summary, a wealth of previous research findings indicates a significant correlation between higher SHR and increased risk of short-term and long-term adverse outcomes in specific patient populations.

In this study, we only found a significant association between SHR in females and all-cause mortality as well as diabetes-related mortality. A meta-analysis of 87 studies previously demonstrated that metabolic syndrome is associated with an increased risk of CVD, with estimated cardiovascular risk consistently higher in women compared to men, particularly in terms of all-cause mortality [19]. The average age of females in this study was 48.19 years, potentially representing the perimenopausal period where estrogen levels are unstable. Estrogen and estradiol can enhance insulin sensitivity and reduce insulin resistance. Additionally, estradiol can directly act on arterial endothelium, altering endothelium-dependent and calcium-dependent processes [20–21]. Therefore, higher SHR may disrupt hormonal balance in females, impairing the normal biological function of insulin. In the population aged ≥ 65 years (elderly), the prevalence of comorbidities is higher. Univariate analysis revealed correlations between each comorbidity and all-cause mortality, indicating that an increased proportion of comorbidities may reduce the contribution of SHR to all-cause mortality. This could explain why higher SHR is not associated with an increased risk of all-cause mortality and diabetes mellitus mortality in the population aged ≥ 65 years.

The results of mediation analysis showed that part of the adverse effect of SHR on all-cause mortality was through its effect on NEU and AST, and part of the beneficial effect was through hemoglobin, RBC, albumin and ALT, among which hemoglobin had the largest beneficial intermediary effect. Interestingly, hemoglobin has an inhibitory effect on the relationship between SHR and all-cause mortality. Early studies have shown that individuals with structural abnormalities of hemoglobin generally exhibit lower insulin resistance compared with individuals with normal hemoglobin levels [22], a result that may constitute a supporting factor for the results of our mediation analysis. However, further studies are needed to confirm the potential relationship between SHR and hemoglobin.

### Impact on clinical practice

By exploring the correlation between SHR and all-cause mortality and diabetes mellitus, the results suggest that SHR can be used as a good predictive indicator. Through the use of online risk calculator, it can help investigators quickly judge the prognostic risk of follow-up individuals. Risk factors can be adjusted and treated in a timely manner to improve outcomes.

### Research limitations

This study has several limitations. First of all, this is a retrospective study, and the results cannot clarify the causal relationship. Prospective studies are needed to obtain more information for verification. Secondly, despite adjusting for covariates and subgroup analysis, it is still impossible to control all potential confounding factors, and causal inference may be limited. Third, because participants were primarily from the United States, the generalizability of the results to other countries may be limited.

## Conclusion

In conclusion, SHR may serve as a predictor of all-cause mortality and diabetes mortality in the US general population, particularly among those aged < 65 years and among women. However, multicenter, prospective studies are still needed to verify this result.

### Electronic supplementary material

Below is the link to the electronic supplementary material.

Supplementary Material 1

Supplementary Material 2

Supplementary Material 3

Supplementary Material 4

Supplementary Material 5

Supplementary Material 6

Supplementary Material 7

## Data Availability

Publicly available datasets were analyzed in this study. This data can be found here: https://wwwn.cdc.gov/nchs/nhanes/Default.aspx.
