# Supplementary figures and images for: Association between stress hyperglycemia ratio and diabetes mellitus mortality in American adults: a retrospective cohort study and predictive model establishment based on machine learning algorithms (NHANES 2009–2018)
Source: Diabetol Metab Syndr. 2024 Apr 2;16:79. doi: 10.1186/s13098-024-01324-w (PMC10986058; doi:10.1186/s13098-024-01324-w)

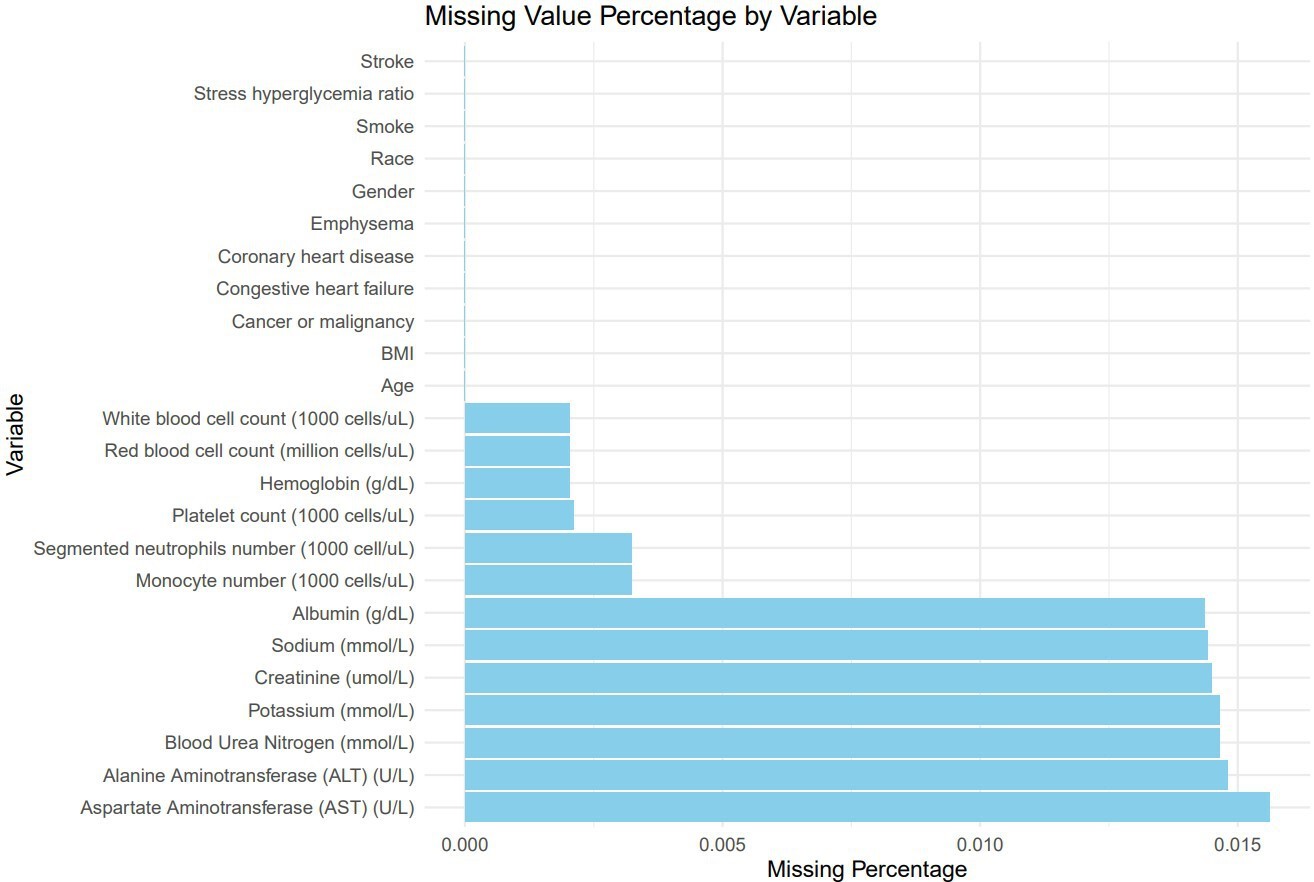

Supplement: Supplementary file 1 — Supplementary Material 1 [file 13098_2024_1324_MOESM1_ESM.jpeg]

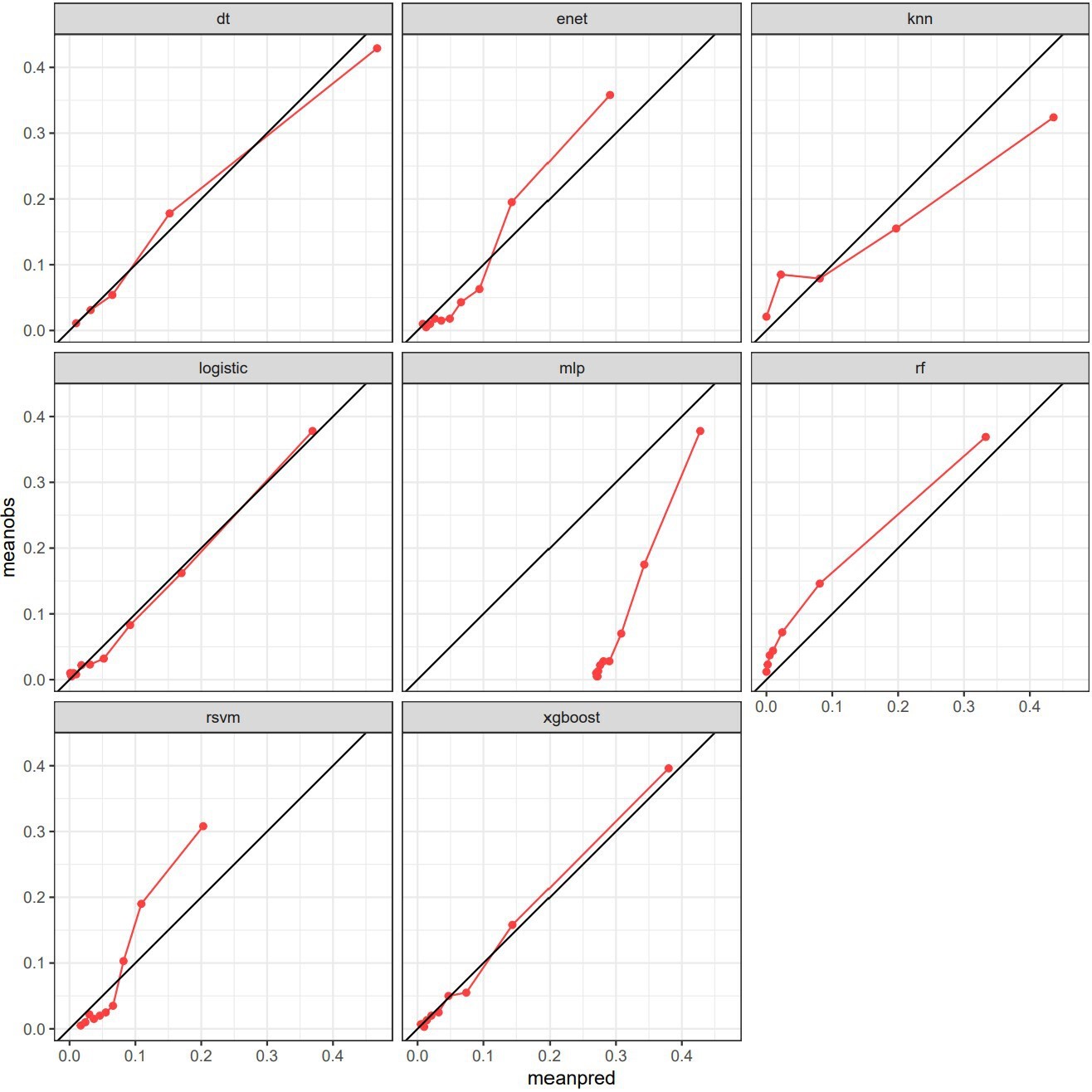

Supplement: Supplementary file 2 — Supplementary Material 2 [file 13098_2024_1324_MOESM2_ESM.jpeg]

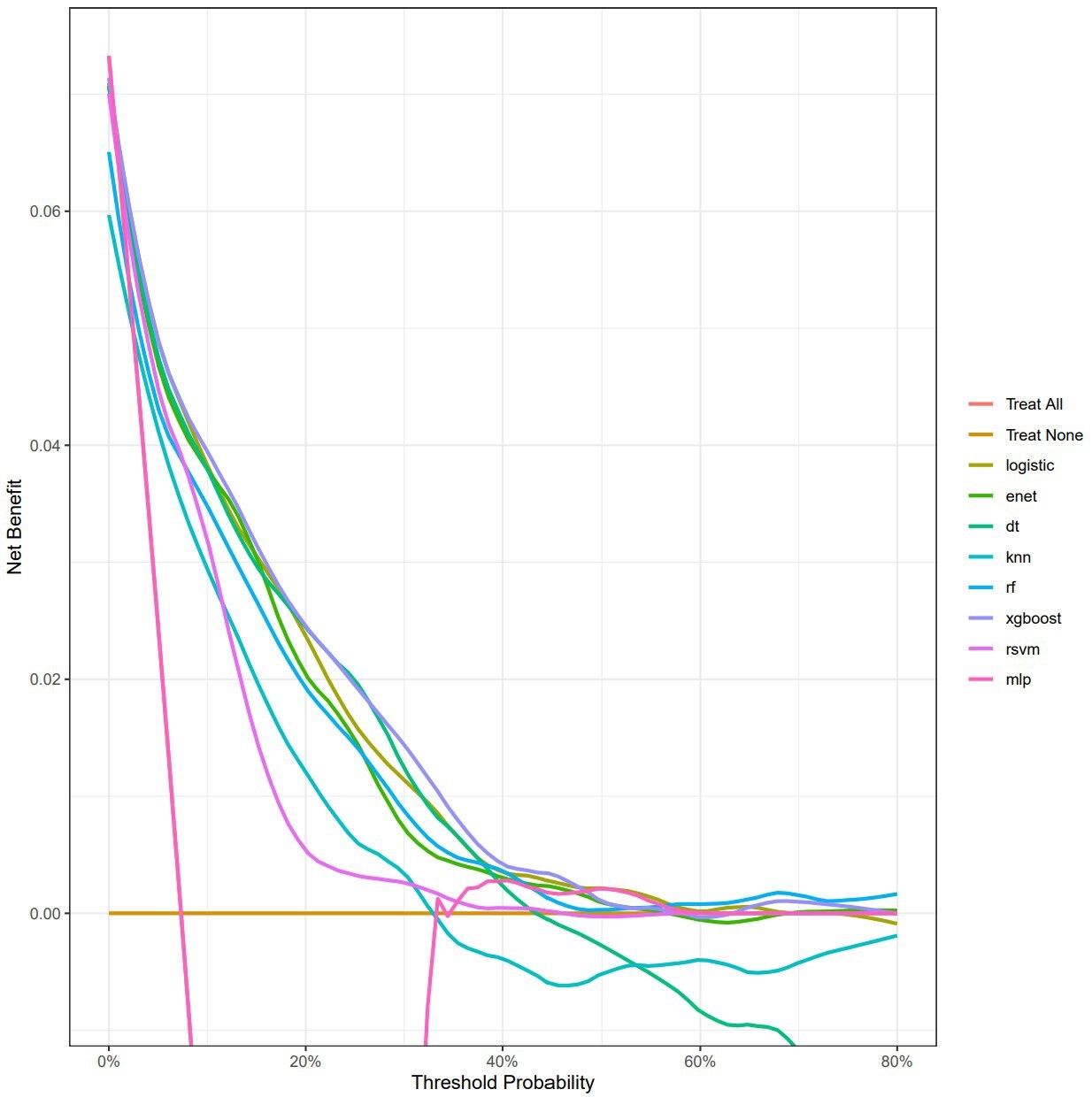

Supplement: Supplementary file 3 — Supplementary Material 3 [file 13098_2024_1324_MOESM3_ESM.jpeg]
