# Supplementary material for: Association between stress hyperglycemia ratio and diabetes mellitus mortality in American adults: a retrospective cohort study and predictive model establishment based on machine learning algorithms (NHANES 2009–2018)
Source: Diabetol Metab Syndr. 2024 Apr 2;16:79. doi: 10.1186/s13098-024-01324-w (PMC10986058; doi:10.1186/s13098-024-01324-w)
Supplement: Supplementary file 4 — Supplementary Material 4 [file 13098_2024_1324_MOESM4_ESM.docx]

| **Variables** | **VIF** |
| --- | --- |
| SHR | 1.065756 |
| AGE | 1.455982 |
| BMI | 1.262228 |
| SMOKE | 1.135525 |
| Monocyte number | 1.532671 |
| Segmented neutrophils number | 3.199919 |
| Hemoglobin | 2.744747 |
| Platelet count | 1.246056 |
| Red blood cell | 2.296937 |
| White blood cell | 3.818833 |
| Blood Urea Nitrogen | 1.736807 |
| Creatinine | 1.534481 |
| Albumin | 1.445392 |
| Aspartate Aminotransferase | 2.078710 |
| Alanine Aminotransferase | 2.244835 |
| Potassium | 1.119916 |
| Sodium | 1.053146 |
| Gender | 1.772098 |
| Race | 1.067446 |
| Congestive heart failure | 1.175943 |
| Coronary heart disease | 1.158475 |
| Stroke | 1.073354 |
| Emphysema | 1.051115 |
| Cancer or malignancy | 1.099128 |

Table S1 Variance inflation factor between variables
