# Supplementary material for: Association between stress hyperglycemia ratio and diabetes mellitus mortality in American adults: a retrospective cohort study and predictive model establishment based on machine learning algorithms (NHANES 2009–2018)
Source: Diabetol Metab Syndr. 2024 Apr 2;16:79. doi: 10.1186/s13098-024-01324-w (PMC10986058; doi:10.1186/s13098-024-01324-w)
Supplement: Supplementary file 5 — Supplementary Material 5 [file 13098_2024_1324_MOESM5_ESM.docx]

Table S2. Univariate and Multivariate COX Regression Analysis (Age Group)

| Characteristic | Age group | | | p-value^2^ |
| --- | --- | --- | --- | --- |
|  | Overall, N = 13,315^1^ | < 65, N = 10,233^1^ | ≥ 65, N = 3,082^1^ |  |
| Congestive heart failure |  |  |  | <0.001 |
| Yes | 438 (3%) | 157 (2%) | 281 (9%) |  |
| No | 12,877 (97%) | 10,076 (98%) | 2,801 (91%) |  |
| Coronary heart disease |  |  |  | <0.001 |
| Yes | 533 (4%) | 166 (2%) | 367 (12%) |  |
| No | 12,782 (96%) | 10,067 (98%) | 2,715 (88%) |  |
| Stroke |  |  |  | <0.001 |
| Yes | 497 (4%) | 205 (2%) | 292 (9%) |  |
| No | 12,818 (96%) | 10,028 (98%) | 2,790 (91%) |  |
| Emphysema |  |  |  | <0.001 |
| Yes | 254 (2%) | 116 (1%) | 138 (4%) |  |
| No | 13,061 (98%) | 10,117 (99%) | 2,944 (96%) |  |
| Cancer or malignancy |  |  |  | <0.001 |
| Yes | 1,195 (9%) | 484 (5%) | 711 (23%) |  |
| No | 12,120 (91%) | 9,749 (95%) | 2,371 (77%) |  |
