# Supplementary material for: Association between stress hyperglycemia ratio and diabetes mellitus mortality in American adults: a retrospective cohort study and predictive model establishment based on machine learning algorithms (NHANES 2009–2018)
Source: Diabetol Metab Syndr. 2024 Apr 2;16:79. doi: 10.1186/s13098-024-01324-w (PMC10986058; doi:10.1186/s13098-024-01324-w)
Supplement: Supplementary file 6 — Supplementary Material 6 [file 13098_2024_1324_MOESM6_ESM.docx]

Table S3: Univariate and multivariate analysis of variables (Cox regression)

|  | Univariable | | | | | Multivariable | | | | |
| --- | --- | --- | --- | --- | --- | --- | --- | --- | --- | --- |
| Characteristic | N | Event N | HR^1^ | 95% CI^1^ | p-value | N | Event N | HR^1^ | 95% CI^1^ | p-value |
| SHR | 13,315 | 987 | 2.06 | 1.36, 3.12 | <0.001 | 13,315 | 987 | 1.47 | 1.03, 2.10 | 0.034 |
| Age | 13,315 | 987 | 1.09 | 1.09, 1.10 | <0.001 | 13,315 | 987 | 1.08 | 1.07, 1.09 | <0.001 |
| BMI | 13,315 | 987 | 1.00 | 0.99, 1.01 | 0.768 | 13,315 | 987 | 0.99 | 0.98, 1.00 | 0.035 |
| Monocyte (1000 cells/uL) | 13,315 | 987 | 1.55 | 1.44, 1.67 | <0.001 | 13,315 | 987 | 1.29 | 1.02, 1.64 | 0.032 |
| NEU (1000 cell/uL) | 13,315 | 987 | 1.08 | 1.06, 1.09 | <0.001 | 13,315 | 987 | 1.10 | 1.05, 1.15 | <0.001 |
| Hemoglobin (g/dL) | 13,315 | 987 | 0.81 | 0.78, 0.84 | <0.001 | 13,315 | 987 | 0.88 | 0.82, 0.95 | <0.001 |
| PLT (1000 cells/uL) | 13,315 | 987 | 0.99 | 0.99, 1.00 | <0.001 | 13,315 | 987 | 1.00 | 1.00, 1.00 | <0.001 |
| RBC (million cells/uL) | 13,315 | 987 | 0.37 | 0.32, 0.42 | <0.001 | 13,315 | 987 | 0.93 | 0.77, 1.13 | 0.487 |
| WBC (1000 cells/uL) | 13,315 | 987 | 1.04 | 1.03, 1.05 | <0.001 | 13,315 | 987 | 0.99 | 0.95, 1.03 | 0.510 |
| BUN (mmol/L) | 13,315 | 987 | 1.23 | 1.21, 1.24 | <0.001 | 13,315 | 987 | 1.03 | 1.00, 1.06 | 0.029 |
| Creatinine (umol/L) | 13,315 | 987 | 1.00 | 1.00, 1.00 | <0.001 | 13,315 | 987 | 1.00 | 1.00, 1.00 | 0.014 |
| Albumin (g/dL) | 13,315 | 987 | 0.25 | 0.21, 0.29 | <0.001 | 13,315 | 987 | 0.43 | 0.35, 0.54 | <0.001 |
| AST (U/L) | 13,315 | 987 | 1.00 | 1.00, 1.00 | 0.003 | 13,315 | 987 | 1.01 | 1.00, 1.01 | <0.001 |
| ALT (U/L) | 13,315 | 987 | 0.99 | 0.98, 0.99 | <0.001 | 13,315 | 987 | 1.00 | 0.99, 1.00 | 0.185 |
| Potassium (mmol/L) | 13,315 | 987 | 2.87 | 2.44, 3.37 | <0.001 | 13,315 | 987 | 1.09 | 0.93, 1.28 | 0.301 |
| Sodium (mmol/L) | 13,315 | 987 | 1.03 | 1.00, 1.06 | 0.084 | 13,315 | 987 | 0.97 | 0.95, 1.00 | 0.032 |
| Gender |  |  |  |  |  |  |  |  |  |  |
| Male | 6,433 | 578 | — | — |  | 6,433 | 578 | — | — |  |
| Female | 6,882 | 409 | 0.65 | 0.57, 0.73 | <0.001 | 6,882 | 409 | 0.63 | 0.54, 0.73 | <0.001 |
| Smoke |  |  |  |  |  |  |  |  |  |  |
| Every day | 2,034 | 180 | — | — |  | 2,034 | 180 | — | — |  |
| Some days | 502 | 20 | 0.45 | 0.29, 0.72 | <0.001 | 502 | 20 | 0.52 | 0.33, 0.83 | 0.007 |
| Not at all | 3,050 | 383 | 1.50 | 1.26, 1.79 | <0.001 | 3,050 | 383 | 0.54 | 0.44, 0.65 | <0.001 |
| Other | 7,729 | 404 | 0.59 | 0.49, 0.70 | <0.001 | 7,729 | 404 | 0.49 | 0.41, 0.60 | <0.001 |
| Race |  |  |  |  |  |  |  |  |  |  |
| Mexican American | 2,013 | 93 | — | — |  | 2,013 | 93 | — | — |  |
| Non-Hispanic Black | 1,434 | 57 | 0.88 | 0.63, 1.23 | 0.452 | 1,434 | 57 | 0.79 | 0.56, 1.10 | 0.162 |
| Non-Hispanic White | 5,251 | 592 | 2.48 | 1.99, 3.08 | <0.001 | 5,251 | 592 | 1.33 | 1.06, 1.68 | 0.013 |
| Other Hispanic | 2,753 | 192 | 1.66 | 1.29, 2.12 | <0.001 | 2,753 | 192 | 1.17 | 0.91, 1.52 | 0.226 |
| Other Race - Including Multi-Racial | 1,864 | 53 | 0.73 | 0.52, 1.03 | 0.071 | 1,864 | 53 | 0.80 | 0.57, 1.13 | 0.205 |
| Congestive heart failure |  |  |  |  |  |  |  |  |  |  |
| Yes | 438 | 157 | — | — |  | 438 | 157 | — | — |  |
| No | 12,877 | 830 | 0.13 | 0.11, 0.16 | <0.001 | 12,877 | 830 | 0.53 | 0.44, 0.65 | <0.001 |
| Coronary heart disease |  |  |  |  |  |  |  |  |  |  |
| Yes | 533 | 153 | — | — |  | 533 | 153 | — | — |  |
| No | 12,782 | 834 | 0.18 | 0.15, 0.22 | <0.001 | 12,782 | 834 | 0.83 | 0.69, 1.00 | 0.053 |
| Stroke |  |  |  |  |  |  |  |  |  |  |
| Yes | 497 | 135 | — | — |  | 497 | 135 | — | — |  |
| No | 12,818 | 852 | 0.20 | 0.17, 0.24 | <0.001 | 12,818 | 852 | 0.71 | 0.59, 0.87 | <0.001 |
| Emphysema |  |  |  |  |  |  |  |  |  |  |
| Yes | 254 | 90 | — | — |  | 254 | 90 | — | — |  |
| No | 13,061 | 897 | 0.15 | 0.12, 0.19 | <0.001 | 13,061 | 897 | 0.59 | 0.47, 0.75 | <0.001 |
| Cancer or malignancy |  |  |  |  |  |  |  |  |  |  |
| Yes | 1,195 | 233 | — | — |  | 1,195 | 233 | — | — |  |
| No | 12,120 | 754 | 0.29 | 0.25, 0.33 | <0.001 | 12,120 | 754 | 0.89 | 0.76, 1.04 | 0.146 |
