# Supplementary material for: Association between stress hyperglycemia ratio and diabetes mellitus mortality in American adults: a retrospective cohort study and predictive model establishment based on machine learning algorithms (NHANES 2009–2018)
Source: Diabetol Metab Syndr. 2024 Apr 2;16:79. doi: 10.1186/s13098-024-01324-w (PMC10986058; doi:10.1186/s13098-024-01324-w)
Supplement: Supplementary file 7 — Supplementary Material 7 [file 13098_2024_1324_MOESM7_ESM.docx]

Mediation analysis for the associations between SHR and survival

| Independent variable | Mediator | Total effect | | Indirect effect | | Direct effect | | Proportion mediated, % (95% CI) |
| --- | --- | --- | --- | --- | --- | --- | --- | --- |
|  |  | Coefficient (95% CI) | P value | Coefficient (95% CI) | P value | Coefficient (95% CI) | P value |  |
| SHR | Congestive.heart.failure | -406.01480 (-868.82154, -68.32282) | 0.024 | -4.34238 (-14.28410, 20.45923) | 0.784 | -401.67242 (-856.52809, -68.79897) | 0.024 | 1.1 (-7.9, 5.5) |
| SHR | Coronary.heart.disease | -371.98088 (-810.41656, -38.36429) | 0.040 | -14.05719 (-22.42071, 1.42168) | 0.088 | -357.92369 (-799.05721, -29.91944) | 0.044 | 3.8 (-1.4, 15.2) |
| SHR | Stroke | -409.98912 (-880.61950, -65.10158) | 0.024 | -9.02029 (-17.24974, 9.10486) | 0.312 | -400.96883 (-871.59703, -61.57784) | 0.032 | 2.2 (-3.3, 9.4) |
| SHR | Emphysema | -471.95382 (-1024.73001, -81.78695) | 0.024 | -2.08048 (-9.29848, 18.97050) | 0.868 | -469.87334 (-1021.32942, -80.11314) | 0.020 | 0.4 (-5.0, 3.0) |
| SHR | Cancer.or.malignancy | -496.69149 (-1067.84149, -90.66917) |  | -5.01612 (-22.62759, 17.73434) |  | -491.67537 (-1058.19268, -86.21668) |  | 1.0 (-4.6, 8.1) |
| SHR | Age | -1058.08061 (-2812.52468, 21.50813) | 0.060 | 431.88227 (-23.83488, 1015.55385) | 0.068 | -1489.96288 (-3281.43490, -417.29016) | <0.001 | -40.8 (-357.2, 151.1) |
| SHR | BMI | -466.76139 (-1039.03092, -83.09567) | 0.024 | 2.74981 (-10.77525, 16.72719) | 0.644 | -469.51120 (-1040.98377, -83.98082) | 0.024 | -0.6 (-6.8, 3.0) |
| SHR | Monocyte.number..1000.cells.uL. | -468.13735 (-1044.55363, -81.51704) |  | 1.10929 (-14.95188, 26.15167) |  | -469.24664 (-1048.72631, -85.72491) |  | -0.2 (-7.1, 3.6) |
| SHR | Segmented.neutrophils.number..1000.cell.uL. | -480.89272 (-1174.58444, -99.46054) | 0.016 | -30.08500 (-150.01095, -15.93462) | <0.001 | -450.80772 (-1140.41967, -34.30470) | 0.028 | 6.3 (3.0, 46.7) |
| SHR | Hemoglobin..g.dL. | -407.36086 (-926.24736, -72.62504) | 0.020 | 282.10054 (192.78691, 427.06017) | <0.001 | -689.46140 (-1320.27976, -301.54703) | <0.001 | -69.3 (-248.7, -34.1) |
| SHR | Platelet.count..1000.cells.uL. | -522.01898 (-1153.45835, -102.73333) | 0.016 | -120.73844 (-202.92866, -69.05808) | <0.001 | -401.28054 (-982.94867, -0.37621) | 0.052 | 23.1 (10.9, 76.3) |
| SHR | Red.blood.cell.count..million.cells.uL. | -484.96893 (-1059.37837, -72.79497) | 0.020 | 174.97182 (100.18489, 280.98036) | <0.001 | -659.94075 (-1262.98472, -210.69617) | <0.001 | -36.1 (-171.6, -15.3) |
| SHR | White.blood.cell.count..1000.cells.uL. | -472.70136 (-1102.40667, -95.68530) | 0.016 | -8.12907 (-36.62975, 0.15320) | 0.056 | -464.57229 (-1089.01558, -89.54147) | 0.020 | 1.7 (-0.1, 9.5) |
| SHR | Blood.Urea.Nitrogen..mmol.L. | -336.25891 (-856.54098, -9.56943) | 0.048 | -55.67585 (-112.58810, -14.40781) | 0.008 | -280.58306 (-775.71572, 46.14446) | 0.080 | 16.6 (-2.8, 70.6) |
| SHR | Creatinine..umol.L. | -541.90875 (-1048.12890, -91.05924) | 0.008 | -9.46470 (-37.55379, 12.77836) | 0.424 | -532.44404 (-1034.61753, -83.60698) | 0.008 | 1.7 (-3.2, 11.0) |
| SHR | Albumin..g.dL. | -386.90059 (-807.36914, -65.27398) | 0.024 | 171.76689 (109.45696, 253.91049) | <0.001 | -558.66748 (-1014.80227, -218.87620) | <0.001 | -44.4 (-181.8, -20.4) |
| SHR | Aspartate.Aminotransferase..AST...U.L. | -464.52977 (-1023.31540, -77.07593) | 0.028 | -6.94012 (-21.93022, -0.88171) | 0.012 | -457.58965 (-1017.64328, -73.22173) | 0.028 | 1.5 (0.1, 7.9) |
| SHR | Alanine.Aminotransferase..ALT...U.L. | -471.52723 (-1034.61156, -88.27018) | 0.020 | 91.69413 (37.75691, 179.34011) | <0.001 | -563.22136 (-1174.95452, -146.07933) | 0.008 | -19.4 (-74.9, -6.9) |
| SHR | Potassium..mmol.L. | -448.70708 (-1036.03746, -75.73702) | 0.020 | -5.74858 (-43.16032, 27.16190) | 0.800 | -442.95850 (-1037.44698, -72.25417) | 0.024 | 1.3 (-9.4, 15.2) |
| SHR | Sodium..mmol.L. | -472.53169 (-1018.57201, -92.52644) | 0.020 | 15.93403 (-3.57444, 39.53945) | 0.112 | -488.46572 (-1034.33752, -111.37782) | 0.008 | -3.4 (-14.7, 1.1) |
